# Functional characterization of a non-coding RUNX1T1 variant in a kindred with esophageal atresia

**DOI:** 10.1080/15476278.2026.2754587

**Published:** 2026-10-30

**Authors:** Nadav Agam, Sarit Aharoni, Baruch Yerushalmi, Galina Ling, Elad Brav, Katya Oustinov, Vadim Dolgin, Ofek Freund, Amit Safran, Matan M. Jean, Tomer Poleg, Nitza Newman, Zaki Assi, Assaf Ben Zvi, Ramon Y. Birnbaum, Suad Sheikh-Suliman, Uri Abdu, Ohad S. Birk

**Affiliations:** a Morris Kahn Laboratory of Human Genetics, National Institute of Biotechnology in the Negev, Ben Gurion University, Be'er Sheva, Israel; b Faculty of Health Sciences, Ben Gurion University, Be'er Sheva, Israel; c Pediatric Gastroenterology Unit, Saban Children's Hospital, Soroka Medical Center, Be'er Sheva, Israel; d Genetics Institute, Soroka Medical Center, Be'er Sheva, Israel; e Department of Pediatric Surgery, Soroka Medical Center, Be'er Sheva, Israel; f Department of Life Sciences, Ben-Gurion University, Be'er Sheva, Israel; g Danek Gertner Institute of Human Genetics, Sheba Medical Center, Ramat-Gan, Israel

**Keywords:** Esophageal atresia, gain-of-function, gene modifier, regulatory element, RUNX1T1

## Abstract

**Introduction:**

Esophageal atresia (EA) is a congenital foregut malformation with substantial genetic heterogeneity. While coding variants explain some syndromic cases, the molecular basis of non-syndromic EA remains largely unknown. We investigated an Israeli-Bedouin kindred with a high incidence of non-syndromic EA.

**Methods:**

Three of ten affected individuals underwent whole-exome/genome sequencing. Following variant filtering and prioritization using population databases and in silico tools, the candidate non-coding variant underwent Sanger sequencing in all five available affected individuals. Functional effects were assessed by minigene splicing assays, quantitative PCR, dual-luciferase reporter assays, and a fly overexpression model.

**Results:**

Sequencing data variant analysis of 3 affected individuals identified a shared heterozygous deep-intronic RUNX1T1 variant (chr8:91994544G > A, hg38), subsequently verified in the five participating affected individuals. Although incorporated into a rare RUNX1T1 transcript, the variant did not affect splicing. Instead, it increased expression of canonical RUNX1T1 isoforms and enhanced reporter activity, consistent with a regulatory gain-of-function effect. RUNX1T1 ortholog (nvy) global overexpression in Drosophila resulted in morphological alterations of the feeding apparatus, including reduced rigidity and elongation of the labral sense organ.

**Conclusion:**

In a kindred with apparently partially penetrant dominant non-syndromic EA, we identified a novel non-coding *RUNX1T1* variant with functional evidence of a regulatory gain-of-function effect. These findings implicate *RUNX1T1* dysregulation as a contributor to EA pathogenesis and highlight the importance of non-coding regulatory variation in congenital foregut malformations.

## Introduction

Esophageal atresia (EA, OMIM 189960) is a rare congenital malformation, affecting approximately one in 3000 live births.^1^ It is characterized by discontinuity of the esophagus and is frequently accompanied by a tracheoesophageal fistula (TEF), an abnormal connection between the esophagus and trachea. EA is thought to arise from disturbances in early foregut development, during which the respiratory and digestive tracts separate to form the ventral trachea and dorsal esophagus.^2^ ^,^ ^3^ This process involves coordinated dorsoventral patterning and morphogenetic remodeling of the foregut, and disruption of these developmental programs can result in abnormal separation and connectivity of the trachea and esophagus.^4^ According to the Gross classification, EA is categorized based on the presence and location of an associated TEF: type A, EA without TEF (8%); type B, EA with proximal TEF (3%); type C, EA with distal TEF (85%); and type D, EA with both proximal and distal TEFs (>1%). Type E accounts for the remaining cases (4%) and denotes a non-syndromic TEF without EA.^5^

Although EA may be suspected during prenatal ultrasonographic evaluation—particularly when an absent or small stomach is observed in the setting of polyhydramnios—these findings have a low positive predictive value, and may have various etiologies; thus, affected newborns are frequently not diagnosed prenatally.^6^ ^,^ ^7^ In fact, EA may follow an uneventful prenatal course and remain undiagnosed during pregnancy; however, it is associated with an increased fetal loss rate of approximately 3%, compared with approximately 0.5% in the general population.^8^ Notably, EA poses immediate, life-threatening risks after birth, including inability to feed, esophageal strictures and dysmotility, and aspiration pneumonia.^9^ Management is primarily surgical and typically begins with urgent interventions to enable safe nutritional support and to eliminate abnormal connections between the gastrointestinal and respiratory tracts.^9^ Definitive repair, aimed at restoring esophageal continuity and function, is usually performed after clinical stabilization, following detailed anatomical assessment of the specific EA subtype and consideration of patient-specific factors.^9^

Overall, EA and tracheoesophageal fistula represent genetically heterogeneous disorders, with confirmed genetic etiologies identified in only approximately 11%–12% of cases, encompassing chromosomal aneuploidies, copy number variants, and rare monogenic syndromes.^1^ ^,^ ^10^ ^,^ ^11^ Although isolated Mendelian inheritance is observed in a small subset of families, the predominance of sporadic cases, low sibling recurrence risk, increased concordance in monozygotic twins, and marked phenotypic variability argue for a strong genetic basis, but not necessarily monogenic for most affected individuals.^10^ ^,^ ^12^

A unifying feature across genetically resolved cases is gene dosage sensitivity, whereby heterozygous loss-of-function variants or copy number alterations disrupt tightly regulated developmental programs, as exemplified by transcriptional and chromatin regulators such as *SOX2,* ^13^ ^,^ ^14^ *CHD7,* ^11^ ^,^ ^15^ *MYCN* ^16^ and *ZIC3,* ^17^ as well as recurrent copy number variants involving *SHOX*, *ABLIM1*, and *LPP.* ^1^ ^,^ ^10^ ^,^ ^18^ Together with the broad spectrum of anatomical presentations and the low overall diagnostic yield, these observations support a polygenic and multifactorial disease architecture. In this model, several genetic variants, each with its individual effect, acting with variable penetrance, interact with environmental, stochastic, or developmental modifiers to determine disease manifestation. Of note, study of animal models of both xenopus and mice highlighted the transcription factors *SOX2* and *NKX2-1* as markers of esophagus and trachea, respectively.^1^

Here, we investigated an inbred Israeli-Bedouin kindred with an unusually high incidence of non-syndromic EA. Given the familial occurrence of EA within this extended kindred, we performed genomic and functional studies to investigate potential genetic factors contributing to the phenotype.

## Materials and methods

### Study population and clinical characterization

Ten individuals of a large isolated Israeli Bedouin kindred were diagnosed with non-syndromic type-C EA (see Figure 1A and B; III-10, III-13, IV-5, IV-7, IV-8, IV-13, IV-14, V-1, V-2, VI-3). Initial clinical presentations included coughing, choking, cyanosis during feeding, and respiratory distress. Failure of gastric tube placement, followed by the typical coiled tube in the proximal esophagus with a gastric bubble in chest radiography, was highly suggestive of type-C EA and was subsequently confirmed by endoscopic evaluation. Clinical genetic evaluation did not identify additional shared phenotypic features indicative of a recognizable syndromic form of EA; in particular, none of the affected individuals fulfilled clinical criteria for VACTERL association or CHARGE syndrome. No consistent facial dysmorphism or developmental delay/intellectual disability was identified. However, variable cardiac and cerebral anomalies were observed in a subset of affected individuals (see Figure 1B).

**Figure 1.**
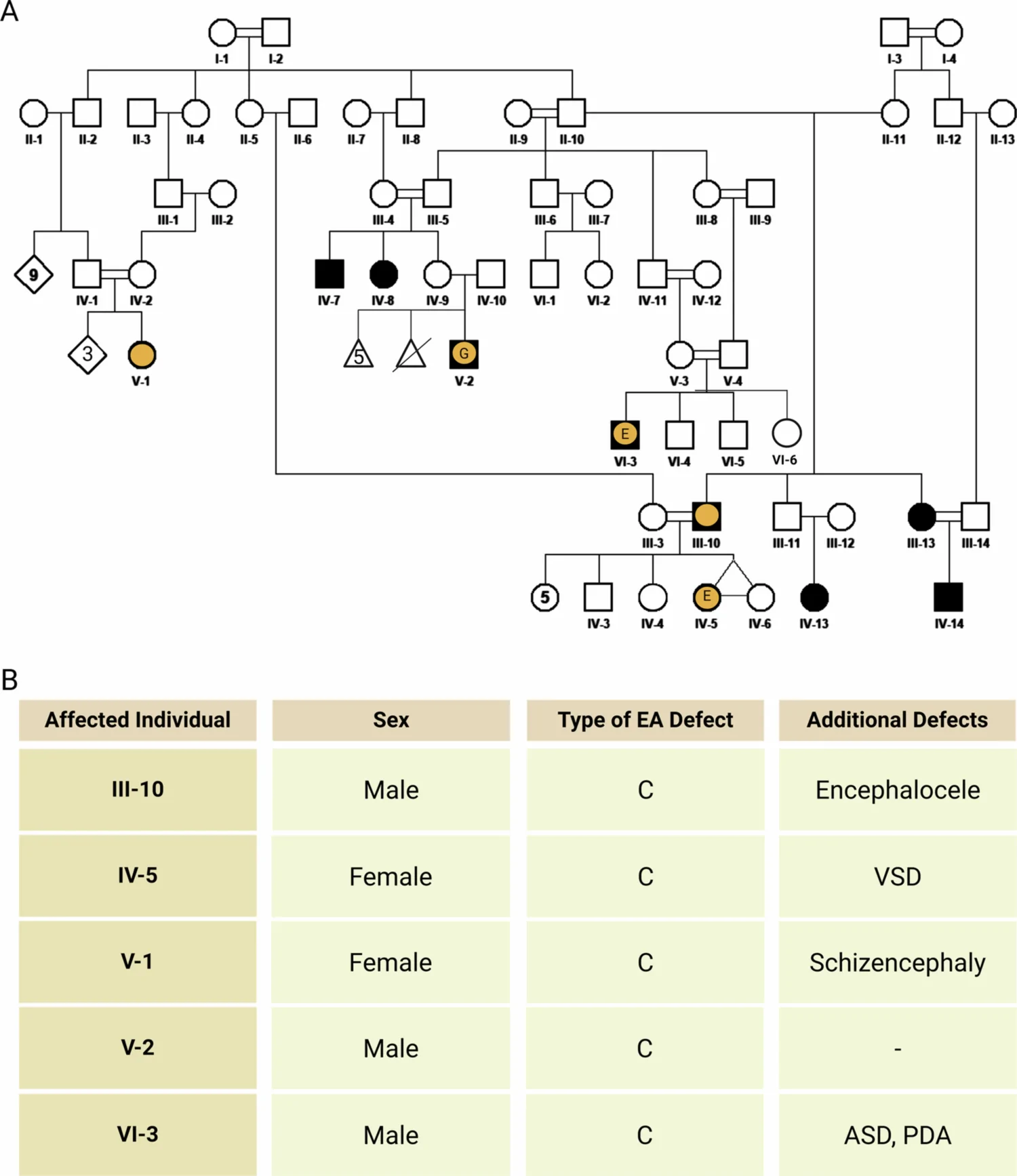
Pedigree and clinical presentation of affected individuals. (A) Pedigree of the studied Bedouin clan. Filled and open symbols denote affected and unaffected individuals, respectively. Yellow circles indicate affected individuals included in the study; E and G denote whole-exome and whole-genome sequencing, respectively. (B) Clinical characteristics of affected individuals. Abbreviations: VSD, ventricular septal defect; ASD, atrial septal defect; PDA, patent ductus arteriosus.

Study participants were recruited following inability to establish a molecular diagnosis through routine clinical genetic evaluation. The phenotypic assessment was conducted by senior pediatric gastroenterologists and geneticists at Soroka Medical Center according to current state-of-the-art clinical standards. All participants or their legal guardians provided written informed consent in accordance with the principles of the Helsinki Declaration, following protocols approved by the Soroka Medical Center Institutional Review Board (IRB, approval No. #5071G) and the Israel National Committee for Human Genetic Studies (approval #920100319).

A checklist for reporting genetic associations, based on the STREGA guidelines, is available in Supplementary materials—Appendix 1.

### Genetic analysis

Genomic DNA was extracted from peripheral blood using the E.Z.N.A.® SQ Blood DNA Kit (OMEGA Bio-tek; SKU D5032-03) according to the manufacturer's instructions. Next generation sequencing (WES; HiSeq 2500, Illumina, San Diego, CA, USA) and whole-genome sequencing (WGS; NovaSeq 6000, Illumina, San Diego, CA, USA) were performed using a paired-end (2 × 100 bp) protocol by Macrogen (Seoul, South Korea). Read alignment, variant calling, and annotation were conducted by the sequencing provider using BWA, GATK, and SnpEff, respectively, and the resulting pipelines were validated in-house. VCF files are available upon request.

Genetic analysis of next-generation sequencing (NGS) data was performed using the VARista platform with default parameters.^19^ Only heterozygous variants shared by all three affected individuals with available whole-exome or whole-genome sequencing data were retained for analysis. Variants observed in the homozygous state or >1% carriership (heterozygotes) rate in GnomAD (https://gnomad.broadinstitute.org/), the Qatar genome project,^20^ or our in-house ethnically matched NGS control cohort were excluded. The remaining variants were subsequently prioritized using the Combined Annotation Dependent Depletion (CADD) scoring system.^21^ High-ranking variants were further assessed for segregation within the studied cohort through Sanger analysis.

Screening for *RUNX1T1* variants in seven unrelated individuals with EA was performed by interrogating NGS data across the *RUNX1T1* locus, including approximately 150 kb of flanking sequence upstream and downstream of the gene. Only variants with a CADD score greater than 15 were considered.

### Alternative splicing assay

To evaluate the impact of the queried variant on alternative splicing, an artificial wild-type transcript was constructed incorporating the first four exons of *RUNX1T1* isoform ENST00000520978.5 (designated as “esoteric”), together with 100–150 bp of flanking intronic sequences (full sequence available in Supplementary materials—Appendix 2). Site-directed mutagenesis was then performed to disrupt the donor splice site of the fourth and terminal exon, thereby enabling proper transcript termination. The resulting sequence was cloned into a pcDNA3.1 expression vector downstream of the CMV promoter using Gibson assembly (full sequence available in Supplementary materials—Appendix 3). The mutant construct was generated by introducing the queried nucleotide substitution into exon 2 using the same site-directed mutagenesis approach. Sequence identity between the wild-type and mutant plasmids, aside from the introduced variant, was confirmed by Sanger sequencing.

For concomitant overexpression of splicing factors, expression plasmids containing the coding sequences of serine/arginine-rich splicing factors *SRSF1*, *SRSF2*, and *SRSF6* were obtained from Addgene (catalog nos. 99021, 44721, and 120419, respectively; Watertown, MA, USA). The mature SRSF5 mRNA was amplified from human cDNA and cloned into a pcDNA3.1 expression vector as described above.

HEK293 cells were cultured in 24-well plates in Dulbecco's modified Eagle medium (DMEM) supplemented with 10% fetal calf serum and 1% penicillin–streptomycin–neomycin. Cells were transfected with the indicated plasmids using PolyJet transfection reagent (catalog no. SL100688; SignaGen Laboratories, Frederick, MD, USA) and incubated for 24 h. Total RNA was isolated using the Direct-zol RNA Miniprep Kit (catalog no. R2053; Zymo Research, Irvine, CA, USA), and cDNA was synthesized using the Verso cDNA Synthesis Kit (catalog no. AB-1453/A; Thermo Fisher Scientific, Waltham, MA, USA). All procedures were performed according to the manufacturers' instructions.

PCR, followed by agarose gel electrophoresis, gel extraction (GeneJET Gel Extraction Kit; catalog no. K0692; Thermo Fisher Scientific, Waltham, MA, USA), and Sanger sequencing, was performed to verify the precise splicing patterns of both the esoteric and canonical isoforms derived from the artificial transcripts. Quantitative PCR was subsequently employed to assess exon 2 inclusion and skipping using two isoform-specific reverse primers in combination with a common forward primer. GAPDH levels were measured and used for normalization (full transcripts and primers sequences are available in Supplementary materials—Appendix 4). All experiments were performed in three independent biological replicates.

### Enhancer reporter assay

To determine whether the genomic region encompassing the queried variant functions as a cis-regulatory element, and to assess the impact of the queried variant on this activity, a dual-luciferase enhancer reporter assay was performed. An approximately 500 bp fragment spanning the variant locus was cloned upstream of a minimal promoter in the M50 Super 8 × TOPFlash reporter construct, driving firefly luciferase expression. Site-directed mutagenesis was used to introduce the queried nucleotide substitution, and both wild-type and mutant constructs were verified by Sanger sequencing (sequences available in Supplementary material—Appendix 5).

HEK293 cells were cultured in 24-well plates in Dulbecco's modified Eagle medium (DMEM) supplemented with 10% fetal calf serum and 1% penicillin–streptomycin–neomycin. Cells were co-transfected with either wild-type, mutant, or empty (no inserted sequence) reporter construct together with the Renilla luciferase normalizing plasmid pGL4.74[hRluc/TK] using PolyJet transfection reagent and incubated for 24 h. Luciferase activity was measured using the Dual-Luciferase Reporter Assay System (catalog no. E1910; Promega, Madison, WI, USA) according to the manufacturer's instructions. Briefly, cells were lysed and firefly luciferase activity was quantified, followed by quenching of firefly luminescence and subsequent measurement of Renilla luciferase activity. Firefly-to-Renilla luminescence ratios were calculated to normalize reporter activity and used as a quantitative measure of enhancer function. All experiments were performed in three independent biological replicates.

### Generation of an in vivo fly model

The following transgenic flies were used: UAS-*nvy* (*nervy*, the fly ortholog of *RUNX1T1* #600314, Bloomington stock center.^22^ Ubiquitous expression was performed with *actin5C*-GAL4. *Actin*-GAL4 line was used as control. Experimental and control pharate specimens were prepared for scanning electron microscopy (SEM; Verios XHR 460L) according to standard protocols prior to imaging. Flies were fixed and dehydrated by immersing them in increasing ethanol concentrations (30%, 70%, and twice in 100%; 10 min each). The flies were then completely dehydrated using increasing hexamethyldisilazane (HMDS) concentrations in ethanol (30%, 70%, and twice in 100%; 90 min each). The samples were air-dried overnight, placed on stubs, and coated with gold. The specimens were examined with a scanning electron microscope (SEM; Verios XHR 460L). Following initial characterization of the phenotype in a preliminary experiment, morphological assessment of subsequent independent experimental cohorts was performed blinded to experimental group.

## Results

### Clinical characterization

Among the ten individuals with EA identified across multiple branches of the extended kindred, five participated in the clinical and molecular study. All five participating affected individuals had type-C EA, with variable associated congenital abnormalities observed in a subset of individuals (Figure 1). No additional shared syndromic phenotype was identified among the affected individuals. Notably, individual V-2 has no living siblings; the parents reported five spontaneous miscarriages occurring at approximately 9–10 weeks of gestation, as well as one elective termination of a pregnancy due to fetal acrania (see Figure 1A). However, no phenotypic or genetic information was available for these pregnancies, precluding assessment of their relationship to EA or to the identified RUNX1T1 variant. Moreover, the occurrence of EA across multiple branches of the pedigree, rather than preferentially within this branch, provides no evidence that the increased number of miscarriages in it is associated with the EA phenotype.

Chromosomal microarrays of all five affected individuals available for testing were normal. Five of the ten affected individuals were available for further molecular genetic studies (Figure 1; III-10, IV-5, V-1, V-2, VI-3). The distribution of affected individuals within the pedigree was most consistent with an autosomal dominant mode of inheritance with incomplete penetrance, estimated at approximately 40% (four affected individuals—III-10, IV-5, V-1, and VI-3—among a total of 20 siblings). This interpretation was supported by several observations. First, an affected individual (IV-13) was born to a clan member father and an unrelated (Arab Bedouin) unaffected mother, arguing against a recessive inheritance model. Second, one affected individual (IV-5) was an identical twin—confirmed by SNP array analysis (data not shown)—of an unaffected sibling and had eight additional unaffected siblings, including one diagnosed with non–cystic fibrosis meconium ileus. Finally, in several nuclear families (e.g., V-1 and IV-5), the number of affected offspring was substantially lower than expected under a fully penetrant dominant model, further supporting incomplete penetrance.

### Genetic analysis

Whole exome and whole genome sequencing data of 3 affected individuals were analyzed. The affected individuals selected for WES/WGS were chosen based on their relationships within the pedigree, with the aim of minimizing their expected genetic similarity and thereby reducing the number of shared variants requiring prioritization. No variants were identified in any of the genes previously associated with EA or TEF. We then searched for shared coding variants using WES data from individuals IV-5 and VI-3. As this analysis yielded no convincing candidate variants, the analysis was subsequently expanded to non-coding variation by performing WGS in individual V-2 and identifying variants shared among the three sequenced affected individuals. Once a defined set of shared candidate non-coding variants had been established, additional genome-wide sequencing of the remaining participating affected individuals was not considered necessary; candidate variants were instead evaluated by Sanger sequencing in all five participating affected individuals.

Notably, no homozygous or compound heterozygous variants were shared by two or more of the affected individuals. Following initial filtering, 25 heterozygous variants shared by those three affected individuals were retained (list of variants available in Supplementary materials—Appendix 6). Eight of those variants exhibited CADD scores exceeding 15. Among them, a deep-intronic, relatively conserved (PhyloP of 4.15) variant in *RUNX1T1* (hg38: chr8:91994544G > A; rs112217168) achieved the highest ranking. *RUNX1T1* was prioritized in part because of previous evidence implicating the gene in gastrointestinal development, including the occurrence of variable midgut atresia in *Runx1t1*-knockout mice.^23^ The heterozygous variant was verified in all five affected individuals participating in the study using Sanger sequencing (see Figure 2A). Although the variant is located deep within an intron in the vast majority of RUNX1T1 isoforms, it is incorporated into exon 2 of the distinct esoteric isoform ENST00000520978.5, as presented in the GTEx portal (https://gtexportal.org/home/, see Figure 2B). While in silico splicing analysis using SpliceAI (https://spliceailookup.broadinstitute.org/) predicted a largely benign effect on splicing, evaluation with ESEfinder 3.0 (https://esefinder.ahc.umn.edu/cgi-bin/tools/ESE3/esefinder.cgi) indicated disruption of a putative exonic splicing enhancer. Although the genomic region surrounding the variant is not annotated as a regulatory element in the GeneHancer database, this possibility was supported by its relatively high evolutionary conservation and the presence of a pronounced signal in the ReMap Project meta-track within the UCSC Genome Browser (https://genome.ucsc.edu/, see Figure 2C). These observations therefore warranted functional assessment of the variant's impact on alternative splicing between this isoform and canonical RUNX1T1 transcripts.

**Figure 2.**
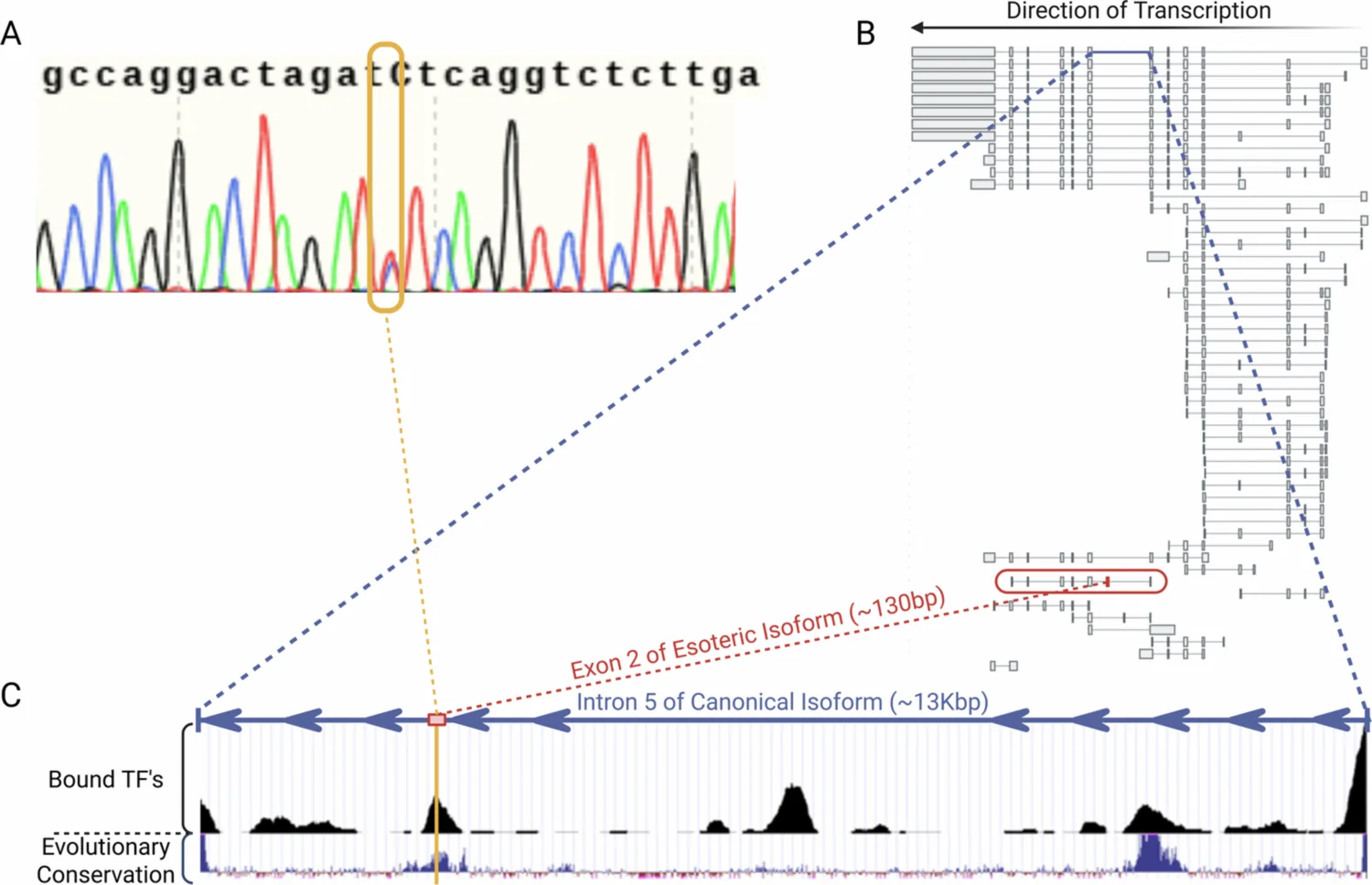
Genetic characterization of the g.8:91994544G > A (hg38) variant. (A) Sanger sequencing chromatogram of an affected individual demonstrating heterozygosity of the G > A substitution. (B) Schematic representation of alternatively spliced *RUNX1T1* isoforms based on GTEx annotations; the queried esoteric isoform is highlighted in red. (C) Schematic view of the genomic regulatory landscape across intron 5 of the canonical *RUNX1T1* isoform. Arrowheads indicate the direction of transcription. The black meta-track denotes experimentally validated transcription factor binding sites, and the dark-blue meta-track indicates evolutionary conservation across vertebrate species (ReMap Project and PhyloP, respectively; visualized using the UCSC Genome Browser). The red rectangle marks exon 2 of the esoteric isoform, and the yellow line indicates the position of the queried variant.

#### The g.8:91994544G > A variant upregulates transcription of canonical *RUNX1T1* isoforms

Expression plasmids (wild-type or mutant) containing the first four exons of the *RUNX1T1* transcript ENST00000520978.5 (referred to as the “esoteric” isoform), together with their flanking intronic sequences, were co-transfected into HEK293 cells along with selected serine/arginine-rich splicing factors (SRSFs, see Figure 3A). Notably, exon 2—containing the queried variant—is unique to the esoteric isoform, whereas exons 1, 3, and 4 are shared with all canonical RUNX1T1 isoforms (see Figure 2B). To assess whether the variant alters alternative splicing between the two isoforms, quantitative PCR was performed.

**Figure 3.**
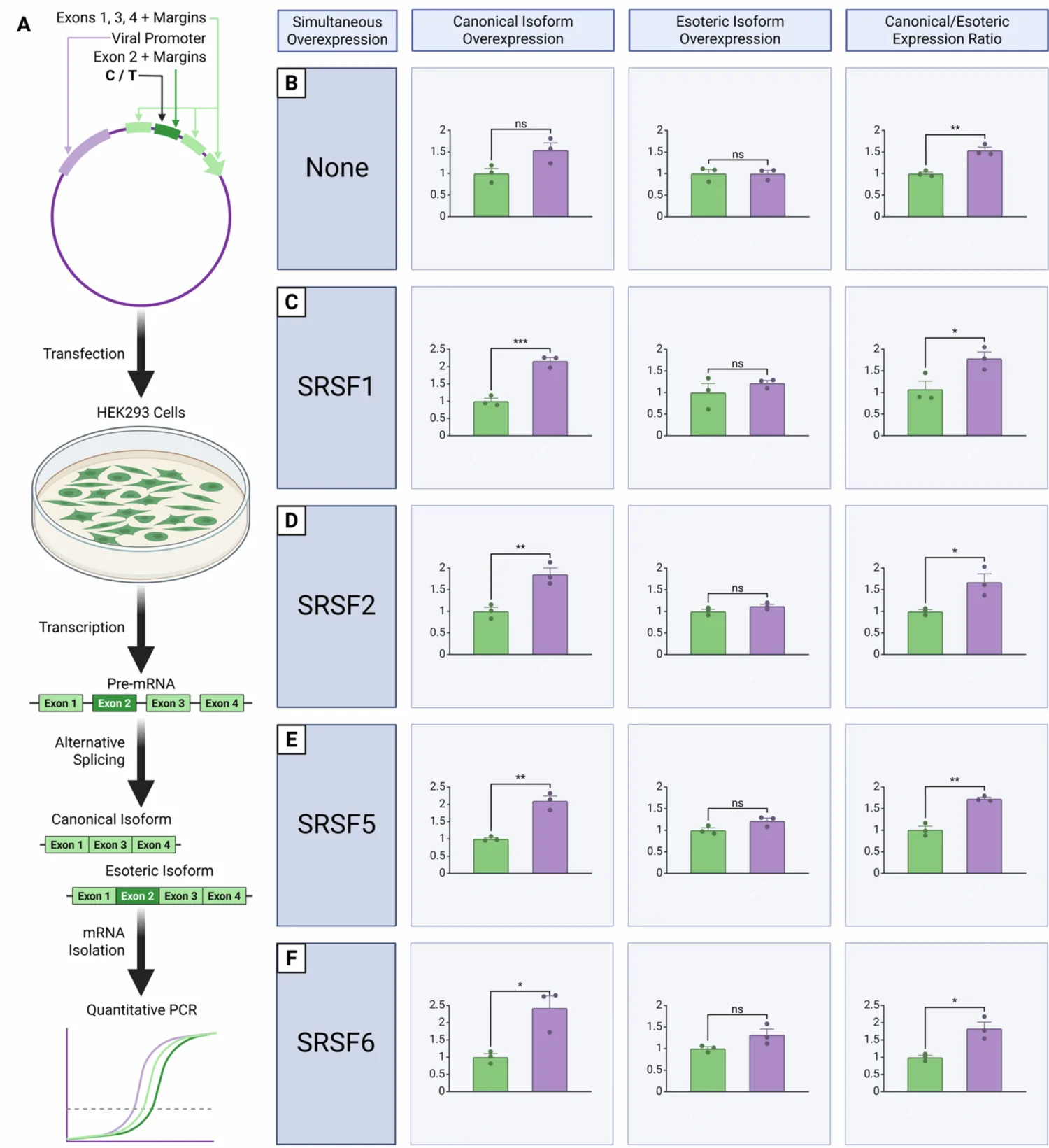
The g.8:91994544G > A Variant Induces Upregulation of the Canonical *RUNX1T1* Isoform. (A) Schematic representation of the experimental setup. (B–F) Quantification of the canonical and esoteric RUNX1T1 isoforms in wild-type versus mutant genotypes, with (C–F) or without (B) simultaneous overexpression of serine-arginine spicing factors: SRSF1 (C), SRSF2 (D), SRSF5 (E), and SRSF6 (F). All data presented as the fold change of the mutant group, compared to the wild-type: green and purple denotes wild-type and mutant genotypes, respectively; individual replicate values, mean, and standard error of the mean (95% confidence interval) appear in darker color. In all comparisons, *n* = 3; unpaired student's *t*-test was performed: ns, *, **, and *** denote nonsignificant, *p*-value < 0.05, *p*-value < 0.01, and *p*-value < 0.001, respectively.

Quantitative assessment of the esoteric isoform revealed no effect of the queried variant, as expression levels remained unchanged in mutant constructs under dominant overexpression of all examined splicing factors (see Figure 3B–F, individual values available in Supplementary materials—Appendix 8). In contrast, expression of the canonical *RUNX1T1* isoform was significantly increased (approximately 2-fold) in the mutant group across all splicing factor conditions. As transcript levels of neither isoform were reduced, these findings indicate that the variant does not impair splicing. Instead, the data suggest a gain-of-function regulatory effect on canonical *RUNX1T1* transcription, prompting further investigation into its impact on overall *RUNX1T1* expression.

#### The g.8:91994544G > A variant has a gain-of-function effect on a cis-regulatory element in which it resides

The queried variant, together with its surrounding ~0.5 kb genomic sequence, was cloned upstream of a minimal promoter driving a luciferase reporter to evaluate the region's enhancer activity and to determine whether introduction of the variant alters this regulatory function (see Figure 4A).

**Figure 4.**
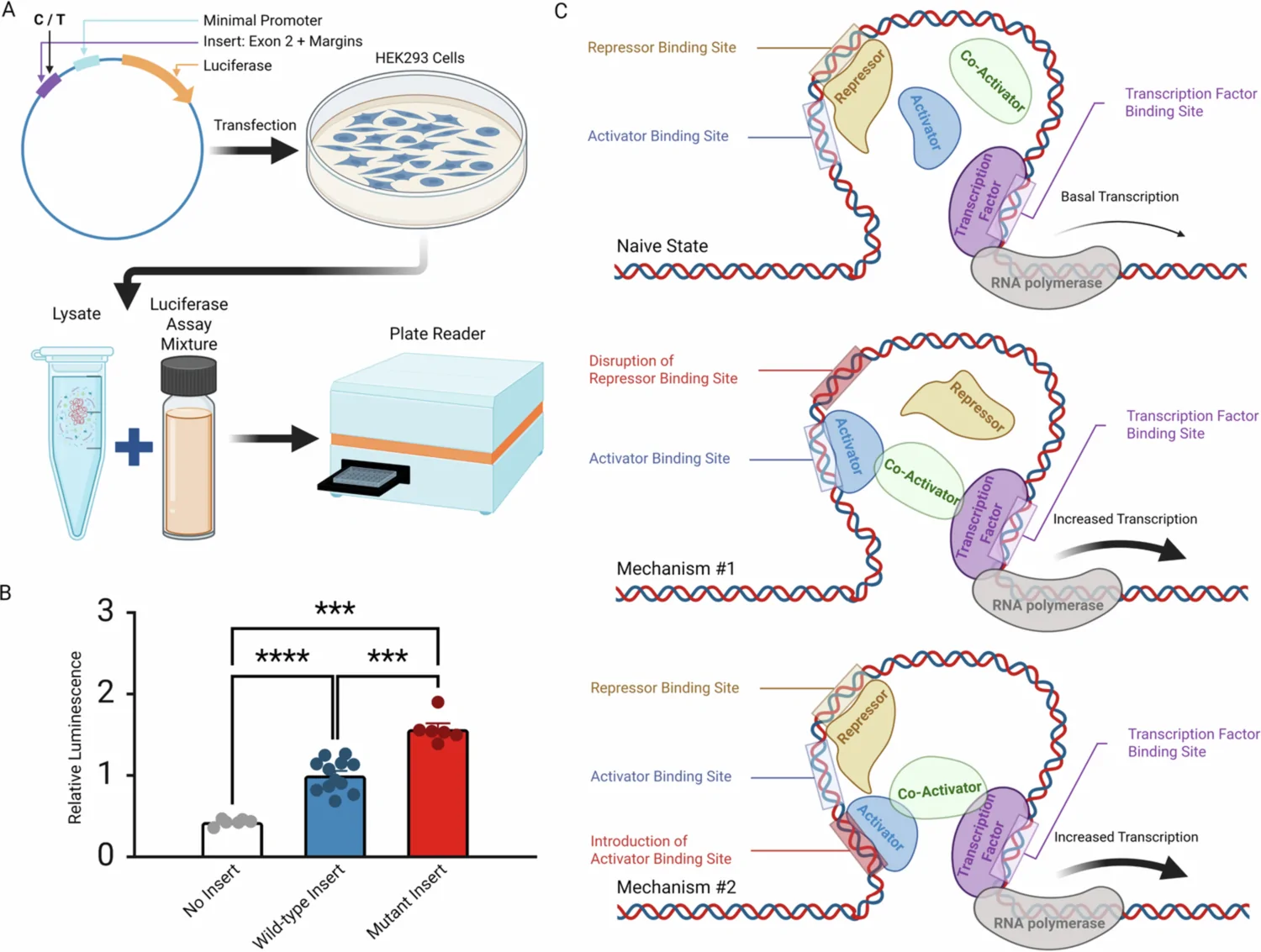
The g.8:91994544G > A variant is located within an enhancer and enhances its activity. (A) Schematic representation of the experimental setup. (B) Dual-luciferase enhancer reporter assay, comparing the transcriptional activity of the No Insert (*n* = 6), wild-type (*n* = 12), and mutant (*n* = 6) groups. Welch's one-way ANOVA test with Games–Howell multiple comparisons was performed. Individual replicate values and standard error of the mean (95% confidence interval) appear in darker color. ***, and **** denote *p*-value < 0.001, and *p*-value < 0.0001, respectively. (C) Mechanisms for mutation-mediated gain-of-function effect in a cis-regulatory element.

The genomic region encompassing the variant exhibited regulatory activity, as it drove significantly higher transcriptional output compared with the minimal promoter alone (see Figure 4B, individual values available in Supplementary materials—Appendix 9). Consistent with the quantitative PCR findings, introduction of the queried variant resulted in a further significant increase in transcriptional activity.

### Global *nvy* overexpression in drosophila results in altered feeding apparatus morphology

Overexpression of the Drosophila *RUNX1T1* ortholog *nvy* using *actin5C*-GAL4 construct showed that larval and pupal development progressed without overt abnormalities; however, they failed to eclose and were found dead within the pupal cases despite reaching morphologically mature adult stages. Gross examination under light microscopy revealed no obvious external structural defects. However, closer examination of the anterior rostral region in mutant flies by scanning electron microscopy revealed a lax, less rigid appearance with altered surface texture of the feeding apparatus (proboscis), including the rostrum, haustellum, and labella, compared with controls. (see Figure 5A–D). Quantitative morphometric analysis further demonstrated that the labral sense organ (LSO), a sensory component of the feeding apparatus, was significantly elongated in the mutant group (see Figure 5E–H).

**Figure 5.**
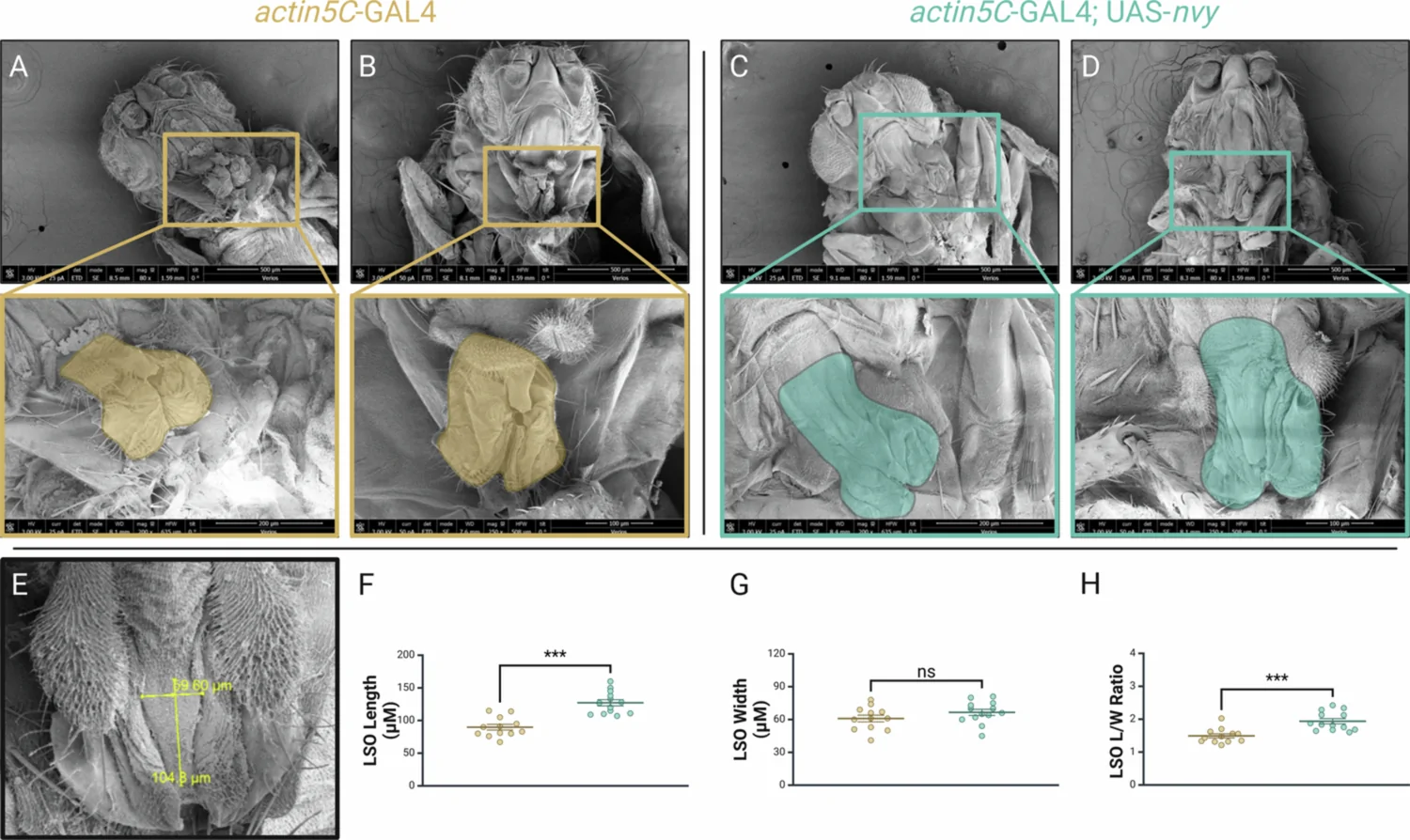
Ubiquitous overexpression of *nvy* (the *RUNX1T1* ortholog) in flies results in altered proportions of structures in their feeding apparatus. (A–D) Pharate adults from *actin5C-GAL4* (A–B, mustard, *n* = 12) or *actin5C-GAL4*; UAS-*nvy* (C–D, turquoise, *n* = 13) genotypes were extracted from their pupal case and examined using SEM. Upper pictures show zoom-out (80× magnification); lower pictures show zoom-in (A, C – 200× magnification, B, D – 50× magnification). Colored polygons delineate the feeding organ: rostrum, haustellum, labella, labial palps, and Labral sense organ (LSO). (E) A representative zoom-in picture of an *actin5c-GAL4* pharate, demonstrating measurements of LSO length and width. (F–H) Comparisons of LSO length (F), Width (G), and length/width ratio (H) in *actin5C-GAL4* x *actin-GAL4* (mustard) and *actin5C-GAL4*; UAS-*nvy* (turquoise) F1 offspring flies. In all comparisons, a Mann–Whitney U Test was performed: ns and *** denote nonsignificant and *p*-value < 0.001, respectively. Mean and standard error of the mean (95% confidence interval) appear in darker color.

## Discussion

The Bedouin population in southern Israel is highly inbred within isolated clans,^24^ leading to augmented allele frequencies of variants that may be absent or exceedingly rare in global reference databases. Consequently, certain traits and diseases occur at higher frequencies in these communities, necessitating adapted clinical indices of suspicion, diagnostic workups, and the use of ethnically matched control datasets in genetic analyses. The queried partially penetrant, autosomal dominant, disease-modifying variant described here exemplifies these population-specific genetic dynamics,^25^ allowing novel insights into molecular pathways of disease and normal development. Notably, it is classified as variant of unknown significance (VUS), according to the ACMG/AMP framework (full description available in Supplementary materials—Appendix 6). The classification exemplifies the recognized limitations of the current framework in interpreting non-coding regulatory variants.^26^

Here, we report an association between a non-coding RUNX1T1 variant and EA in extended inbred kindred. Human genetic and functional in vitro studies demonstrate that the identified deep-intronic RUNX1T1 variant lies within a regulatory element and upregulates RUNX1T1 expression. Together with the observed phenotypes following manipulation of RUNX1T1 orthologs in animal models, these findings implicate RUNX1T1 as a candidate gene that may contribute to the etiology of EA. However, the available data does not establish a causal relationship between the identified variant and EA. Furthermore, although all five participating affected individuals had type-C EA, this is the most common anatomical subtype of EA, and the present study does not provide evidence for a specific relationship between RUNX1T1 and type-C EA. Notably, cerebral malformations were observed in two affected individuals, although their relationship to the EA phenotype or to the identified RUNX1T1 variant remains unknown.

Both cardiovascular and central nervous system anomalies have been described in individuals with EA.^27^ ^,^ ^28^ Although cerebral malformations were observed in two of the five participating affected individuals in the present kindred, the small size and familial nature of this cohort preclude meaningful comparison of the prevalence of associated anomalies with that reported in larger EA populations. Thus, the present data do not establish whether cerebral or cardiovascular anomalies are enriched in this kindred, and the cerebral abnormalities observed may represent co-existing congenital anomalies rather than manifestations related to EA or the identified RUNX1T1 variant.

The high prevalence of autosomal-recessive disorders in consanguineous Bedouin populations has been well documented.^24^ Given the high degree of consanguinity within this population, and within this extended kindred in particular, the cerebral and cardiovascular abnormalities observed in individual family members may represent manifestations of co-existing genetic conditions rather than features related to EA or to the identified heterozygous *RUNX1T1* variant. To assess a possible association between the EA phenotype and other clinical findings, systematic evaluation of the phenotypic status of unaffected members of the extended kindred would be required, including members of family branches in which no individuals with EA were identified. As such an evaluation was not feasible in the current study, we make no claims regarding an association between the *RUNX1T1*-associated EA phenotype, and other medical conditions.

Our functional analyses indicate that the queried g.8:91994544G > A variant resides within a previously unrecognized cis-regulatory element and does not affect splicing or the coding sequence. Accordingly, while we clearly show that the variant upregulates RUNX1T1 expression, one cannot rule out the possibility that this variant may also influence the expression of neighboring genes, including *PIP4P2, OTUD6B, LRRC69, SLC26A7* and *TRIQK*, through local regulatory effects. However, none of those neighboring genes have an expression pattern or known function of likely relevance to the EA phenotype.

Although midgut atresia in *Runx1t1*-deficient mice provided biological support for considering *RUNX1T1* during variant prioritization, it should not be interpreted as evidence that midgut atresia and EA share a common developmental mechanism. The foregut and midgut undergo distinct regional specification and morphogenesis, and the morphology of midgut atresia does not recapitulate that of type-C EA.^3^ ^,^ ^4^ Thus, the murine phenotype supports a broader role for *RUNX1T1* in gastrointestinal development but does not, by itself, establish a mechanistic link between *RUNX1T1* dysfunction and EA.

A similar caution applies to interpretation of the Drosophila phenotype. The elongated labral sense organ (LSO) observed following *nvy* overexpression should not be interpreted as an anatomical or developmental correlate of human EA. The LSO is a sensory component of the Drosophila feeding apparatus and is not homologous to the vertebrate esophagus. Notably, neuronal abnormalities have been described as potentially contributing to the pathophysiology of EA, including evidence of abnormal development of esophageal innervation.^29^ Of potential relevance, cerebral malformations were also observed in two affected individuals in the present kindred, although their relationship to the EA phenotype or to the identified RUNX1T1 variant remains unknown. Interestingly, manipulation of the Drosophila counterparts of two prominent genes whose loss of function causes EA in mouse models, *SOX2* and *SHH* (*SoxN* and *hh*, respectively), does not result in an EA-like phenotype or an analogous esophageal malformation.^14,30–32^ However, *hh* signaling has been shown to directly influence the development and patterning of the Drosophila mouthparts, including the labial structures.^33^ The cause of lethality associated with global *nvy* overexpression in flies remains uncertain. Although they reached morphologically mature adult stages, they failed to eclose and died within the pupal cases; therefore, the observed feeding-apparatus abnormalities cannot be assumed to account for the lethal phenotype. Given the global nature of *nvy* overexpression, additional developmental or physiological effects in other tissues, especially the nervous system, may have contributed to the failure to eclose, and this study do not establish a causal relationship between the lethality and either the feeding-apparatus morphology or the human EA phenotype.

*RUNX1T1* encodes a transcriptional corepressor and has been extensively studied predominantly in the context of acute myeloid leukemia (AML), where it is involved in t(8;21), the most frequent chromosomal translocation associated with AML, generating the pathogenic RUNX1–RUNX1T1 fusion protein.^34^ Of note, no cases of AML or other hematologic malignancies were reported among the participating affected individuals or within their extended family branches upon specific inquiry. Moreover, we are not aware of evidence establishing an association between EA and AML, and population-based studies of individuals with EA have not demonstrated an increased overall cancer risk.^35^ Given the limited size of the present kindred, however, our findings do not permit assessment of whether the identified *RUNX1T1* regulatory variant has any relevance to AML susceptibility, or if it may constitute a potential target for precise intervention.

While ample studies have focused on the AML-causing RUNX1–RUNX1T1 fusion protein, RUNX1T1 has been studied also in its native form: It assembles into a complex with RBPJ (Recombination Signal Binding Protein for Immunoglobulin Kappa J Region) and SHARP (SPEN/RITA), repressing transcription target genes of the Notch signalling pathway.^36^ RUNX1T1 has also been suggested to act as an epigenetic modifier.^37^

Genes associated to date with EA, most notably *SOX2, CHD7, MYCN* and *NKX2-1*, are mostly relevant to syndromic forms of the phenotype: *SOX2* dominant variants have been shown to cause partial penetrance of EA in the context of a syndrome of microphthalmia/optic nerve hypoplasia with or without central nervous system and genital phenotypic features; Heterozygous variants in *CHD7* have been shown to cause variable expression of EA/TE-fistula as part of CHARGE syndrome; Dominant loss of function variants in *MYCN* can cause partial penetrance of EA as part of Feingold syndrome, with microcephaly and intellectual disability, as well as variable ocular and limb malformations.^38^ Variants in *NKX2-1*, are known to cause chorea, hypothyroidism and neonatal respiratory distress in humans; however, its loss-of-function in mice results in abnormal foregut development characterized by failure of tracheoesophageal septation, producing a common lumen connecting the pharynx to the stomach rather than the anatomically distinct trachea and esophagus.^39^ Although this phenotype has been described as resembling tracheoesophageal fistula, it does not recapitulate the anatomy of human EA and is more consistent with a severe defect of tracheoesophageal separation. Notably, in all these monogenic syndromic forms of EA, the phenotype is of partial penetrance, as it is in our cohort of non-syndromic *RUNX1T1*-related EA. The partial penetrance/variable expression evident in this study is also well in line with the causative variant being in an enhancer, as enhancer germline variants have been often associated with variable penetrance/expression.^40^ ^,^ ^41^

Based on the known genetic modifiers of syndromic EA, one can attempt to delineate molecular pathways/interactions related to causation of EA by gain-of-function of *RUNX1T1*. Two of the most well-established genes that have been confirmed to cause EA when mutated are *SOX2* and *NKX2-1.* ^1^ Study of animal models have shown their marking of the specification of endodermal epithelium in the dorsal-esophageal and ventral-respiratory domains, respectively. These processes have been demonstrated to constitute an interaction between the endoderm and surrounding splanchnic mesoderm. Moreover, iPSCs derived from esophageal atresia patients revealed SOX2 dysregulation at the anterior foregut stage, as well as abnormal expression of NKX2-1 in mature esophageal organoids.^42^ Notably, *Sox2* has been demonstrated to be a Notch target gene in mice, being directly activated by Notch intracellular domain and repressed by the RBPJ complex.^43^ As RUNX1T1 assembles into a complex with RBPJ and SHARP and represses transcription target genes of the Notch signalling pathway,^36^ it is plausible that RUNX1T1 gain-of function down-regulates SOX2 expression, driving causation of EA. Consistent with the foregut-separation defect described above, *Nkx2-1* has a critical role in tracheoesophageal morphogenesis; tracheal separation has been shown to involve *Nkx2-1*-mediated repression of Efnb2 and regulation of endodermal cell sorting.^39^ ^,^ ^44^ Additionally, overexpression of Notch intracellular domain was reported to reduce the spatial domains in which *nkx2-1* is expressed in Zebrafish.^45^ Thus, as RUNX1T1 represses transcription target genes of the Notch signalling pathway,^36^ it is plausible that its gain of function might affect *nkx2-1* expression.

Possible molecular pathways through which RUNX1T1 gain of function might enhance causation of EA might involve also *CHD7* and *MYCN*: protein-protein interaction screens have demonstrated direct interaction between CHD7 and RBPJ, known to form a complex with RUNX1T1 and SHARP repressing transcription target genes of the Notch signalling pathway.^36^ Of interest, RUNX1T1 also directly affects MYCN activity: the transcriptional co-repressor Runx1t1 is essential for MYCN-driven neuroblastoma tumorigenesis.^46^ More relevant to possible molecular pathways of EA: MYCN has been shown to drive increased RUNX1T1 translation.^46^ While the above data suggest possible molecular pathways linking altered RUNX1T1 activity to EA, the precise mechanisms by which RUNX1T1 may contribute to the morphogenesis of foregut-derived structures remain to be elucidated. One potential link may involve its previously described role in mesodermal and vascular development, where RUNX1T1 regulates VEGFA, BMP4, and TGF-β2 expression.^47^ Of potential relevance, signaling from the surrounding splanchnic mesoderm, including BMP signaling, contributes to dorsoventral patterning and tracheoesophageal specification of the foregut. Thus, altered RUNX1T1-dependent mesodermal signaling represents one possible mechanism by which RUNX1T1 dysregulation could influence foregut development, although this remains speculative.^48^

Over the past several decades, advances in human genetics have progressively expanded our ability to assign molecular, biological, and ultimately clinical significance to non-coding genetic variations. Interpretation has shifted from the identification of relatively straightforward alterations, such as aneuploidies or coding variants, toward a more comprehensive understanding of how non-coding single-nucleotide variants, indels, or structural variants influence gene integrity, expression, and translation. This transition has been enabled by the stepwise development of large-scale genomic databases and increasingly integrative tools for variant annotation, visualization, and functional inference. Together, these resources have facilitated the exploration of regulatory genomic regions and the genetic architecture of more subtle, complex traits.

Our study leverages several state-of-the-art genomic, bioinformatic, and functional approaches to elucidate the molecular consequences of a queried variant, thereby exemplifying the potential of integrative variant interpretation. It underscores the added value of close collaboration between clinicians and basic researchers, combining detailed phenotyping with computational analysis and functional validation. The relatively small cohort size and the partial penetrance of the autosomal dominant inheritance pattern limit the strength of the proposed gene–disease association. Nevertheless, irrespective of whether the variant acts as a contributing genetic factor to EA in otherwise healthy individuals, our data demonstrate a clear upregulatory effect on gene expression, leading to the identification of a previously unannotated enhancer or cis-regulatory element. This effect may result from disruption of a repressor binding site, introduction of an activator binding motif, or enhancement of the affinity of an activator site to its corresponding binding factor (see Figure 4C). Notably, while gain-of-function variants in regulatory elements are a recognized mechanism in human disease, they remain relatively rare.^49^

The spatiotemporal expression pattern governed by the identified regulatory element, and the way the queried variant alters this activity, remain to be determined. An in vivo transgenic enhancer reporter assay in zebrafish did not demonstrate detectable activity for either the wild-type or mutant constructs (data not shown). Further investigation using mammalian model systems may be required to elucidate the precise spatiotemporal regulatory activity of this element. Nevertheless, available functional evidence suggests that this element is predominantly active across multiple cancer cell lines, as well as in several embryonic tissues (ReMap Project visualized using the UCSC Genome Browser). Notably, regulatory activity has been observed in at least three patient-derived leukemic cell lines, including Kasumi-1, a well-established AML model harboring the t(8;21) translocation and expressing the RUNX1–RUNX1T1 fusion protein. These observations warrant further investigation into the potential role of this regulatory element in oncogenesis, particularly in AML, and whether it may represent a previously unrecognized target for gene-level precision therapeutic strategies.

In conclusion, our findings identify RUNX1T1 as a candidate genetic factor that may have a contributory role in the development of EA. We delineate a previously unannotated intronic cis-regulatory element whose activity is enhanced by the queried variant, displaying a regulatory gain-of-function mechanism. The downstream developmental consequences of altered RUNX1T1 expression and their relationship to EA require further investigation. These findings add to the growing body of genetic evidence implicating regulators of foregut morphogenesis in the pathogenesis of EA.

### Limitations

Recruitment and genetic sampling within this extended kindred were challenging because of limited access to medical and genetic services and limited willingness to participate in research-oriented genetic testing beyond clinically indicated evaluation. Consequently, only five of the ten identified individuals with EA participated in the molecular study. The distribution of affected individuals across the pedigree was not consistent with a straightforward autosomal-recessive inheritance pattern, despite the high degree of consanguinity, and suggested a dominant pattern with incomplete penetrance. Given the limited willingness of family members to undergo genetic testing, recruitment efforts therefore prioritized affected individuals for variant identification. Only a small number of unaffected relatives were potentially accessible for sampling, which would not have allowed a reliable estimate of variant penetrance, with any practical meaning.

## Supplementary Material

Supplementary Material RUNX1T1 in EA Supplementary Materials Uploaded again-.docx

## Data Availability

Codes, packages, and experimental protocols are detailed in the Methods sections and references or links to their sources. Genomic data of affected individuals (SNP arrays, WGS) or any additional information or questions will be provided upon request.
